# Activation of Resolution Pathways to Prevent and Fight Chronic Inflammation: Lessons From Asthma and Inflammatory Bowel Disease

**DOI:** 10.3389/fimmu.2019.01699

**Published:** 2019-07-23

**Authors:** Cindy Barnig, Tjitske Bezema, Philip C. Calder, Anne Charloux, Nelly Frossard, Johan Garssen, Oliver Haworth, Ksenia Dilevskaya, Francesca Levi-Schaffer, Evelyne Lonsdorfer, Marca Wauben, Aletta D. Kraneveld, Anje A. te Velde

**Affiliations:** ^1^Department of Chest Disease, Strasbourg University Hospital, Strasbourg, France; ^2^Equipe d'accueil 3072, University of Strasbourg, Strasbourg, France; ^3^Immunowell Foundation, Utrecht, Netherlands; ^4^Human Development and Health, Faculty of Medicine, University of Southampton, Southampton, United Kingdom; ^5^National Institute for Health Research Southampton Biomedical Research Centre, University Hospital Southampton NHS Foundation Trust and University of Southampton, Southampton, United Kingdom; ^6^UMR 7200 CNRS/Université de Strasbourg, Laboratoire d'Innovation Thérapeutique and LabEx MEDALIS, Faculté de Pharmacie, Strasbourg, France; ^7^Division of Pharmacology, Utrecht Institute for Pharmaceutical Sciences, Faculty of Science, Utrecht University, Utrecht, Netherlands; ^8^Nutricia Research, Utrecht, Netherlands; ^9^Biochemical Pharmacology, William Harvey Research Institute, Bart's School of Medicine and Queen Mary University of London, London, United Kingdom; ^10^Division of Pharmacology, Faculty of Science, Utrecht Institute for Pharmaceutical Sciences, Utrecht University, Utrecht, Netherlands; ^11^Pharmacology and Experimental Therapeutics Unit, Faculty of Medicine, School of Pharmacy, Institute for Drug Research, The Hebrew University of Jerusalem, Jerusalem, Israel; ^12^Department of Biochemistry & Cell Biology, Faculty of Veterinary Medicine, Utrecht University, Utrecht, Netherlands; ^13^Institute for Risk Assessment Sciences, Faculty of Veterinary Medicine, Utrecht University, Utrecht, Netherlands; ^14^Amsterdam UMC, Tytgat Institute for Liver and Intestinal Research, University of Amsterdam, AGEM, Amsterdam, Netherlands

**Keywords:** resolution, inflammation, immune fitness, eicosanoids, asthma, chronic inflammatory bowel disease

## Abstract

Formerly considered as a passive process, the resolution of acute inflammation is now recognized as an active host response, with a cascade of coordinated cellular and molecular events that promotes termination of the inflammatory response and initiates tissue repair and healing. In a state of immune fitness, the resolution of inflammation is contained in time and space enabling the restoration of tissue homeostasis. There is increasing evidence that poor and/or inappropriate resolution of inflammation participates in the pathogenesis of chronic inflammatory diseases, extending in time the actions of pro-inflammatory mechanisms, and responsible in the long run for excessive tissue damage and pathology. In this review, we will focus on how resolution can be the target for therapy in “Th1/Th17 cell-driven” immune diseases and “Th2 cell-driven” immune diseases, with inflammatory bowel diseases (IBD) and asthma, as relevant examples. We describe the main cells and mediators stimulating the resolution of inflammation and discuss how pharmacological and dietary interventions but also life style factors, physical and psychological conditions, might influence the resolution phase. A better understanding of the impact of endogenous and exogenous factors on the resolution of inflammation might open a whole area in the development of personalized therapies in non-resolving chronic inflammatory diseases.

## Introduction

Inflammation is part of the normal response of the host to invasion by harmful microorganisms or to tissue injury (1). The acute inflammatory response is initiated within minutes of recognition of a danger signal, and begins with an onset phase coordinated by several families of chemokines, cytokines, eicosanoids, proteases, vasoactive amines, neuropeptides and neurotransmitters, and other pro-inflammatory mediators produced by resident immune and structural cells in the injured/infected tissue, which is followed by a rapid influx of granulocytes from blood to the tissue inflammatory site (2). Self-amplifying networks of pro-inflammatory pathways perpetuate leukocyte recruitment and activation.

**Figure 1:**
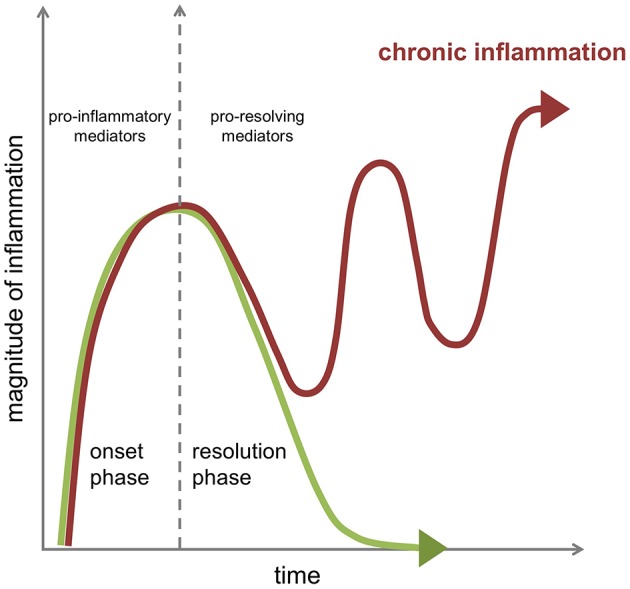
Dynamics of the inflammatory response in chronic inflammation. The acute inflammatory response is a highly coordinated sequence of events characterized by an onset phase coordinated by several families of chemokines, cytokines, and pro-inflammatory mediators that is followed in health by an active resolution phase brought about by the engagement of specific cellular mechanisms under the control of several pro-resolving mediators to promote resolution of the tissue inflammation as well as healing and repair. A failure in pro-resolving pathways can extend in time the actions of pro-inflammatory mechanisms resulting in prolonged or chronic inflammation with recurrent exacerbations.

In a state of immune fitness, the inflammatory response is contained in time and space, and is programmed to resolve, i.e., return from the infected or injured state to a “healthy” state corresponding to that of pre-inflamed tissue. Formerly considered as a passive process, the natural resolution of acute inflammation is now known as an active host response, with highly coordinated cellular and molecular events with release of anti-inflammatory cytokines, loss of receptors for pro-inflammatory signals, and production of a wide range of pro-resolving mediators including recently uncovered specialized pro-resolving lipid mediators (SPMs) that enable restoration of tissue homeostasis (3). A failure in pro-resolving pathways may extend in time the actions of pro-inflammatory mechanisms resulting in prolonged or chronic inflammation with recurrent exacerbations, responsible in the long run for excessive tissue damage and pathology (Figure 1).

Poor and/or inappropriate resolution of inflammation has indeed emerged as a critical process in the pathogenesis of numerous chronic inflammatory and auto-immune diseases including inflammatory bowel diseases (IBD) (such as Crohn's disease and ulcerative colitis) (4). Persistent airway inflammation in chronic lung diseases, such as asthma, may also be due to defects in pro-resolving molecular pathways (5, 6).

The possibility to promote resolution of the inflammatory response as a therapeutic approach has only become apparent in the twenty-first century (7, 8). Better understanding the resolution phase of the inflammatory response and how this process might be influenced by environmental factors might open a whole area of new, affordable, and personalized therapeutic options in chronic inflammatory diseases. This article will first review the main cellular and molecular mechanisms involved in the resolution of inflammation. Finally, we will discuss a series of interventions that can potentially promote resolution with a focus on “Th1/Th17 cell-driven” and “Th2 cell-driven” immune diseases, with IBD and asthma, as relevant examples.

## The Main Determinants of the Resolution Phase

**Figure 2:**
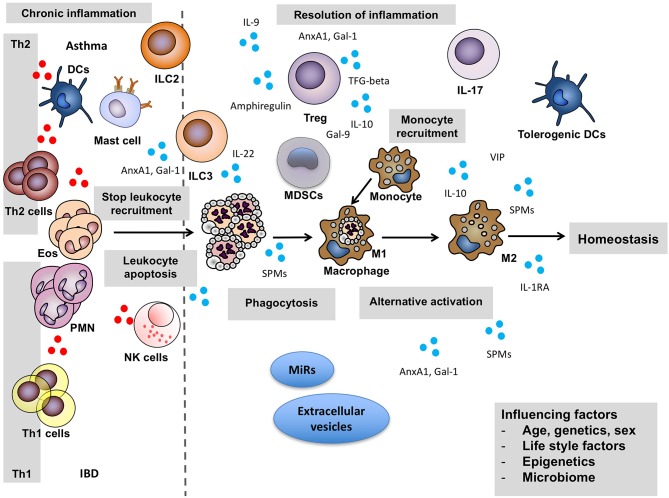
Key cellular actors of resolution. For effective resolution of inflamed tissues to occur and to restore tissue homeostasis, specific cellular mechanisms that are under the control of pro-resolving mediators are enlisted. They promote termination of the inflammatory response and initiate tissue repair and healing. Pro-inflammatory mediators: red circles, pro-resolving mediators: blue circles. Anx, annexin; DCs, Dendritic cells; Eos, eosinophils; Gal, Galectin; IBD, inflammatory bowel disease; IL, interleukin; ILC2, Type 2 innate lymphoid cells; ILC3, Type 3 innate lymphoid cells; MDSCs, Myeloid-derived suppressor cells; MiRs, MicroRNAs; NK, Natural killer; PMN, polymorphonuclear cells; TGF-beta, Transforming growth factor beta; Th1, Type 1 T helper cells; Th2, Type 2 T helper cells; Treg, regulatory T cells; SPMs, specialized pro-resolution lipid mediators; VIP, vasoactive intestinal peptide.

Overall there are two distinct phases in an inflammatory reaction: the initiation of inflammation and the resolution phase (Figure 1). A post-resolution phase of inflammation that links innate and adaptive immune systems has also been described (9, 10). For effective resolution of inflamed tissues to occur and to restore tissue homeostasis, specific cellular mechanisms that are under the control of pro-resolving mediators are enlisted to promote termination of the inflammatory response and initiate tissue repair and healing (Figure 2). Better understanding of how the environment can impact on the resolution of inflammation will lead to an improved understanding of why the chronic inflammatory diseases persist.

### Cells Involved in the Resolution of Inflammation

#### Macrophages

One of the key events in determining the initiation of the resolution phase is the recruitment of non-phlogistic monocytes and their differentiation into macrophages at sites of inflammation. Indeed, central to the successful resolution of inflammation, is the process of local leukocyte clearance by apoptosis and subsequent phagocytosis of the apoptotic cells by surrounding monocyte-derived phagocytes (Figure 2). Engulfment of apoptotic cells signals to the phagocytosing macrophages that the inflammatory response is ending, and alters macrophage mediator production from a predominantly pro-inflammatory (M1) to an anti-inflammatory and pro-resolving phenotype (M2), that further enhances phagocytosis of apoptotic cells and promotes the return to tissue homeostasis (11, 12). This shifting balance between pro-inflammatory M1 and wound-healing M2 macrophages over time is essential for proper resolution of inflammation (13).

#### Regulatory T Cells

Regulatory T (Treg) cells can also play roles in the resolution process, by promoting repair and regeneration of various organ systems and may link innate and adaptive immune systems [for a recent review see (14)]. Treg cells, like T helper (Th) cells, derive from the progenitor CD4^+^ naive T cell. The population of Treg cells consists of thymus-derived Treg cells called natural Treg (nTregs) cells, and Treg cells induced in the periphery or induced Treg (iTregs) cells. Treg cells suppress the activation and function of inflammatory leukocytes, specifically macrophages, through the production of anti-inflammatory cytokines (IL-10 and TGF-β) and by scavenging IL-2 (high expression of IL-2R CD25), signaling of surface molecules, cytolysis, and metabolic control (15, 16).

Treg cells are important players for maintaining homeostatic balance in the intestine [reviewed in (17)]. Acute Treg cell deficiency results in an exacerbated inflammatory immune response toward commensal intestinal bacteria leading to a chronic inflammatory state as found in IBD (18).

Similarly, in mouse models of allergic asthma, resolution of allergic airway inflammation was dependent on CD4^+^CD25^+^Foxp3^+^ expressing Treg cells (19). Accumulation of Treg cells in local draining lymph nodes of the lung correlated with spontaneous resolution of chronic asthma in another murine model (20). Moreover, in the lung, Treg cells have also been described to directly stimulate lung tissue repair, as a consequence of the production of amphiregulin, an autocrine growth factor (21).

#### Innate Lymphoid Cells

Innate lymphoid cells (ILCs) are a large family of cells with various immunological functions (22). They can be classified into different subgroups based on their cytokine production and their expression of key transcription factors, similar to T cell subsets. In various mouse models of asthma and IBD, studies suggest a role for ILCs in the induction of inflammation [for recent reviews see (23, 24)]. Recent evidence suggests a more complex role for these cells, with dual roles in the induction of inflammatory diseases but also the control of chronic inflammation. Type 2 ILC (ILC2) cells demonstrate a flexibility and plasticity dependent on the local microenvironment and can potentially act both as effectors and suppressors [reviewed in (25, 26)]. ILC2 cells, by producing IL-5 and IL-13, promote the development of type 2 allergic inflammation, independent of Th2 cells (9, 27). In contrast, the production of IL-9 by ILC2s was recently reported to mediate resolution of inflammation in a model of chronic arthritis, another chronic non-resolving disease (28). Also, a potential role for ILC2 has been suggested in tissue repair after acute lung injury in a mouse model of H1N1 influenza virus infection through the production of amphiregulin (29).

The same holds true for type 3 ILC (ILC3) cells, the most abundant ILC subtype in the human intestine at steady state (30). ILC3 cells are the main contributors to intestinal IL-22 production, which is a tightly regulated mediator for immune homeostasis in the intestinal tract (31).

NK cells are also members of the ILC family with potential roles of SPM-induced resolution of eosinophilic inflammation in Th2 asthma (32, 33).

#### Myeloid-Derived Suppressor Cells

Myeloid-derived suppressor cells (MDSCs) are a heterogeneous population of cells, consisting of myeloid progenitor cells, and immature macrophages, granulocytes and dendritic cells. These cells are not present in the normal healthy steady state, and appear in pathological situations related to chronic inflammatory situations and stress. Their main function is the suppression of T cell function (34–36).

Recently, a recommendation was published to classify these cells into two different subsets based on their phenotype and function (37). Polymorphonuclear (PMN) and mononuclear (M) MDSCs share several partly overlapping immunosuppressive mechanisms, where inhibition of anti-CD3/CD28-induced T-cell proliferation and IFN-γ production are the general functional tests used for their identification. In general, MDSCs use several mechanisms to carry out their immunosuppressive function. As biomarkers, the expression of various transcription factors and apoptotic regulators (pSTAT3, cEBP/b, S100A8/9) and immune-regulatory genes and molecules (ARG1, NOS2, NOX2, and PNT) are associated with MDSCs and/or PMN-MDSC and M-MDSC subsets (37, 38). These molecules have immunosuppressive effects and negatively regulate T cells by impairing IL-2R signaling pathways, trigger apoptosis (39, 40), and induce Treg cell expansion and IL-10 and TGF-ß production (41, 42). Moreover, MDSCs have a relevant role in resolution of inflammation by efferocytosis of apoptotic neutrophils (35), a process supported in part by IL-10 (42).

### Mediators Participating in the Resolution of Inflammation

During the inflammatory response, diverse mediators are synthesized in a strict temporal and spatial manner to act on specific receptor targets and to actively prevent the overshooting of acute inflammatory mechanisms, and ultimately restore tissue homeostasis. Functionally, these anti-inflammatory and pro-resolving mediators counter-regulate key events of inflammation. Different from solely anti-inflammatory actions, pro-resolving mediators actions typically target specific pro-resolution mechanisms: limitation and/or cessation of neutrophil recruitment; promotion of non-phlogistic monocyte recruitment; induction of neutrophil apoptosis and their subsequent efferocytosis by macrophages, enhancement of efferocytosis, reprogramming of macrophages from classically activated to alternatively activated cells; return of non-apoptotic cells to the blood or egress via the lymphatic vasculature; stimulation of tissue repair and cellular repopulation of the tissue, leading to “adapted homeostasis” [recently reviewed in (43)].

Pro-resolving mediators are diverse in nature, and include SPMs (lipoxins, resolvins, protectins, and maresins), proteins and peptides [annexin A1 (AnxA1), galectins, adrenocorticotropic hormone (ACTH), and IL-10], gaseous mediators including hydrogen sulfide (H2S) and carbon monoxide (CO), nucleotides (e.g., adenosine), as well as neuromodulators released under the control of the vagus nerve such as acetylcholine and neuropeptides released from non-adrenergic non-cholinergic neurons (44). As diverse as their nature is their origin, where mediators of resolution can be produced locally, acting in paracrine and autocrine manners, or produced at distant sites, followed by their systemic release and extravasation to sites of inflammation (43). Below the main pro-resolving mediators will be described.

#### Specialized Pro-resolving Lipid Mediators (SPMs)

**Figure 3:**
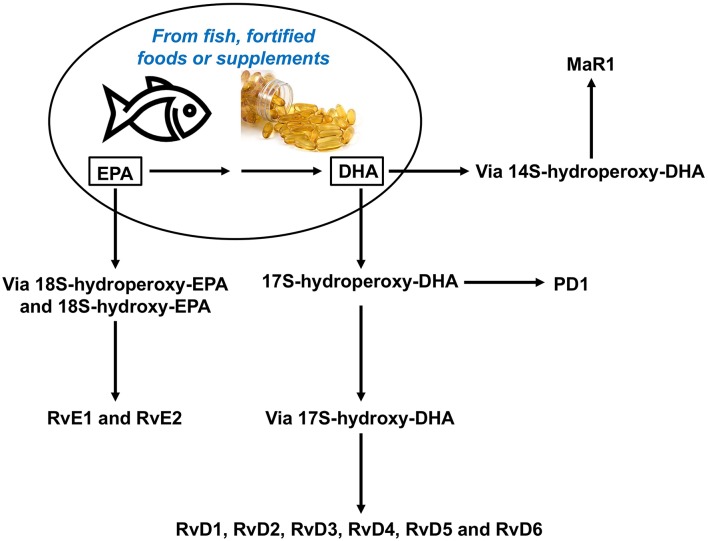
Overview of the pathways for synthesis of resolvins from omega-3 polyunsaturated fatty acids, DHA and EPA. DHA, docosahexaenoic acid; EPA, eicosapentaenoic acid, MaR, maresin; PD, protectin; Rv, resolvin.

Recently, a new array of lipid molecules that function in the resolution of inflammation were elucidated and collectively named SPMs (3, 45, 46). These mediators, such as lipoxins (Lx), resolvins (Rv), protectins (PD), and maresins (Mar), are produced during the inflammatory response and derive from polyunsaturated fatty acids (PUFAs). Whereas, Lx derive from the omega-6 PUFA arachidonic acid, the omega-3 PUFAs eicosapentaenoic acid (EPA) and docosahexaenoic acid (DHA) give rise to Rv, PD, and Mar (Figure 3). More recently SPMs produced from both the omega-6 and omega-3 docosapentaenoic acids have been described (47). The SPMs are produced via biosynthetic circuits engaged during cell–cell interactions including different innate immune cells, for example macrophages or neutrophils, and structural cells at sites of inflammation. SPMs can also been produced through interactions of platelets with leukocytes (48).

**Figure 4:**
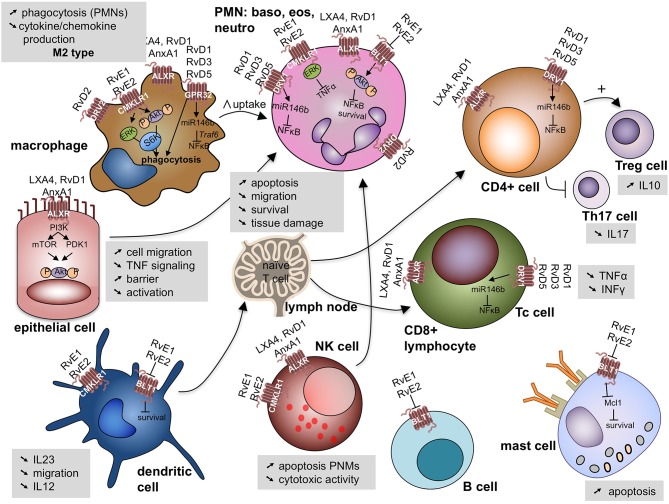
Specialized pro-resolving lipid mediators signal through G-protein coupled receptors on a variety of cell involved (deranged) immune response leading to cell specific responses. Akt, protein kinase B; ALX/FPR2, N-formyl peptide receptor 2—LXA_4_ receptor; AnxA1, annexin A1; BLT1, leukotriene B_4_ receptor 1; CD, cluster domain; CMKLR1, chemokine like receptor 1 or Chemerin Receptor 23; DVR1, RvD1 receptor or G protein coupled receptor (GRP)32; DVR2, RvD2 receptor or GRP18; ERK, extracellular signal regulated kinases; IL, interleukin; INFγ, interferon γ; Mcl-1, anti-apoptotic protein in mast cells; miR, microRNA; mTOR, mammalian target of rapamycin; NK cell, natural killer cell; NFκB, nuclear factor kappa-light-chain-enhancer of activated B cells; P, phosphorylated; PDK1, phosphoinositide-dependent protein kinase 1; PI3K, phosphatidylinositol 3-kinase; PMN, polymorphonuclear cells; Rv, resolvin; LX, lipoxin; S6K, ribosomal protein S6 kinase; Th17 cell: Thelper 17 lympocyte; Treg cell: regulatory T lymphocyte; TNFα, tumor necrosis factor α; Traf6: TNF receptor associated factor 6.

These bioactive lipids display potencies in the nanomolar range, and signal through cognate G-protein coupled receptors (GPR) such as the N-formyl peptide receptor 2 (ALX/FPR2), GPR32, and GPR18 with many cell type-specific actions (49, 50) (Figure 4).

LXA_4_ binds to the ALX/FPR2. This receptor displays diverse ligand affinities that extend beyond interactions with LXA_4_. Indeed, ALX/FPR2 can interact with over 30 ligands with various affinities, and has been identified as the first receptor to engage both bioactive lipids and peptides/proteins, including annexin A1 (50). ALX/FPR2 is widely expressed on human leukocytes, including neutrophils, eosinophils, monocyte-macrophages, T cells, NK cells, and ILC2 cells, as well as on tissue resident cells, such as airway epithelial cells and fibroblasts (33, 51, 52). Its expression is up-regulated by local inflammatory-mediators such as IL-13 and IFN-γ (51, 53).

After initiation of the resolution of inflammation, repolarization by resolvin E1 (RvE1) induces a M2 wound healing-type macrophage (54). In addition, different Rv and Mar interact with ERV1/ChemR23, GPR32 and GPR18 on macrophages to enhance their efferocytosis, phagocytosis and IL-10 transcription (54–61). Other more recently described targets of these mediators are Treg cells and type 2 ILCs (33, 62). SPMs can prevent naïve CD4^+^ T cell differentiation into Th1 and Th17 cells and enhance the generation of Treg cells (63).

Evidence for the functional importance of these lipid mediators in the resolution of inflammation comes from mouse models of diverse inflammatory disorders where SPMs are able to control inflammation, limit tissue damage, shorten resolution intervals, and promote wound healing [for a recent reviews see (6, 16, 64)].

#### Annexin A1

An important mediator of the resolution of inflammation is the glucocorticoid-regulated protein annexin (Anx) A1, also known as lipocortin-1. AnxA1 is highly abundant in myeloid-derived cells such as neutrophils and macrophages, and exerts profound effects on several phases of the resolution of inflammation (65). AnxA1 signals through the FPR2, which also binds the SPMs LxA_4_ and RvD1 (50). Studies in mice indicate that this protein has important modulatory functions in neutrophil trafficking by reducing neutrophil infiltration and activating neutrophil apoptosis. AnxA1 also promotes monocyte recruitment, clearance of apoptotic neutrophils by macrophages and can switch macrophages toward a pro-resolving M2 phenotype (65). Studies have demonstrated that mast cell-derived AnxA1 is important for the cromones-induced inhibition of allergic mast cell degranulation (16).

#### IL-10

IL-10 is a cytokine important in controlling excessive inflammation. It mediates its major functions through inhibition of cytokine production and down-regulating antigen presentation by macrophages, monocytes, and dendritic cells (DCs) and thereby inhibiting adaptive immune cells such as Th2 and Tregs (66–68). IL-10 can also inhibit eosinophilia, by suppression of IL-5 and GM-CSF and by direct effects on eosinophil apoptosis [(69) #1607; (70) #1622].

#### Galectins

Galectins are ß-galactoside-binding lectins produced by, and acting upon, cells of both the innate and adaptive immune systems, modulating multiple processes within the host. Some members of this family of lectins are proposed to play pro-resolving functions, namely Galectin (Gal)-1 and 9. Gal-1 is found in resolving exudates in a murine model of peritonitis induced by zymosan (71), where it stops recruitment of neutrophils and lymphocytes (72, 73). DCs that are differentiated in a Gal-1 rich environment show enhanced regulatory function, reducing the progression of inflammation in a mouse model of multiple sclerosis by promotion of IL-10-mediated T-cell tolerance (74). Gal-1 also induces the conversion of macrophages into a pro-resolving M2 phenotype (75). Gal-3 enhances efferocytosis of apoptotic granulocytes by monocyte-derived macrophages (MDMs) (76). Gal-9 promotes apoptosis of extravasated immune cells including neutrophils and Th1 cells, and is protective in different experimental animal models of chronic auto-immune diseases (77–79). Finally, Gal-1 and Gal-9 promote the generation of Treg cells (80, 81) and induce the production of IL-10 by peripheral blood mononuclear cells from healthy donors (82).

#### ACTH and Melanocortins

Melanocortins, including adrenocorticotrophic hormone (ACTH) and the α, β and γ-melanocyte-stimulating hormone (MSH) are derived from a larger precursor molecule known as the pro-opiomelanocortin (POMC) protein. They exert their numerous biological effects by activating 7 transmembrane GPCR (83). ACTH does not only induce cortisol production, as previously assumed, but also exerts anti-inflammatory actions by targeting melanocortin receptors present on immune cells (84). The protective actions of melanocortins include inhibition of leukocyte transmigration and reduction of pro-inflammatory cytokine production (85, 86). Melanocortins also promote clearance of apoptotic cells (86) and cutaneous wound healing (87).

#### Gaseous Mediators

Carbon monoxide (CO) and hydrogen sulfide (H_2_S) are the best characterized gaseous substances that, in addition to their important roles in physiological and pathophysiological processes, have confirmed pro-resolving actions during inflammatory processes [reviewed in (88)]. H_2_S promotes neutrophil apoptosis and stimulates macrophage phagocytosis (89, 90). CO can inhibit leukocyte migration and reduce pro-inflammatory cytokine production (91). CO has shown therapeutic potential in animal models of acute lung injury (92).

#### Adenosine

Adenosine, is a purine nucleoside generated by the dephosphorylation of adenine nucleotides. In addition to being a potent endogenous physiologic and pharmacologic regulator of many functions, adenosine has pro-resolving mechanisms including inhibition of neutrophil and T cell functions, efferocytosis and macrophage reprogramming [reviewed in (93)].

#### Neuropeptides and Neurotransmitters

It is important to consider that not only immunological mediators, but also factors produced by the nervous system, like neuropeptides and neurotransmitters, contribute to the resolution of inflammation. During an inflammatory response several anti-inflammatory neuropeptides with an immunomodulatory role are produced. One example is vasoactive intestinal peptide (VIP) displaying anti-inflammatory functions in various models of chronic inflammatory disease. VIP impairs the development and infiltration of self-reactive Th1 cells into target organs, as well as the release of inflammatory cytokines and chemokines and the subsequent recruitment and activation of macrophages and neutrophils (94). In addition, VIP stimulates the production of IL-10 and IL-1RA, both important mediators of resolution, and induces the generation of tolerogenic DCs regulating the Th/Treg cells balance (95–99). Very recently, VIP was shown to modulate the differentiation of human macrophages toward the M2 phenotype, which is important in the resolution of inflammation (100).

Another example is the vagal regulation of immune responses, specifically controlling resolution and the production of SPMs (101). Disruption of the vagal system delays the resolution of the inflammatory response upon bacterial peritoneal infections via reduced numbers of group 3 ILCs (102). In macrophages, the nicotine acetylcholine receptor, α7nAChR, mediates anti-inflammatory actions and contributes to the regulation of phagocytosis. Especially M2-type macrophages express this receptor that has a protective and pro-survival role (103) and M2-type macrophages are important producers of protectin conjugates in tissue regeneration (PCTR)1 during resolution (104).

### Other Mediators Participating to the Resolution of Inflammation

#### Anti-inflammatory Cytokines

TGF-β is a potent inhibitor of classical pro-inflammatory macrophage activation (105). TGF-β is also a mediator in critical processes in wound healing, stimulating angiogenesis, fibroblast proliferation, collagen synthesis and deposition and remodeling of extracellular matrix (106, 107). Additionally, TGF-β regulates immune responses through the development and differentiation of Th17 cells and FoxP3^+^ Treg cells (108, 109). TGF-β inhibits the differentiation of T helper subsets as it inhibits the expression of Tbet and GATA3, thereby blocking the differentiation of Th1 and Th2 cells respectively (110, 111).

IL-22 primarily targets non-hematopoietic cells and plays a role in host defense at barrier surfaces where it promotes tissue regeneration (112). IL-22 is produced by Th17 and Th22 cells, ILCs and NKT cells (113). It has different roles in the gastrointestinal tract including tissue regeneration, maintenance of the intestinal barrier and intestinal defense against pathogens (113–116). IL-22 levels are enhanced in the lungs of patients with asthma (117). However, in inducible lung-specific IL-22 transgenic mice, a significant decrease in allergic airway hyperresponsiveness and allergic inflammation occurred indicating an immune modulating effect of IL-22 (118).

IL-1RA (receptor antagonist) is a natural inhibitor of the pro-inflammatory cytokine IL-1 as it functions as an IL-1 receptor competitor (119). It is produced by CD163^+^ wound healing M2 macrophages (120). In IBD, polymorphism in the IL-1RA gene have been demonstrated and an imbalance of IL-1 and IL1RA has been suggested to induce mucosal inflammation associated with IBD (121, 122).

More recently, IL-4 has been reported to induce macrophage proliferation and activation with reduced pulmonary injury after infection with a lung-migrating helminth (123).

#### MicroRNAs

MicroRNAs (MiRs) are small non-coding RNA molecules that can bind to complementary sequences of mRNA molecules thereby regulating/inhibiting post-translational gene expression. MiRs are contributors to the resolution of inflammation by targeting pro-inflammatory genes (124). MiRs 21, 146b, 208a, and 219 are increased during the resolution phase of acute resolving peritonitis in mice (125) and RvD_1_ can regulate expression of these proresolving MiRs (126). MiR-146b down-regulates NF-κB signaling (127), and MiR-219 targets 5-lipoxygenase, with a decreased formation of leukotrienes (19). These results indicate that MiRs actively contribute to resolution of inflammation.

#### Extracellular Vesicles

The paracrine manner of the cellular communication in resolution may be achieved not only by secretion of immune mediators but also through extracellular vesicles. Extracellular vesicles are small membrane vesicles (exosomes, microvesicles, and apoptotic bodies) secreted by all cell types including immune cells in a controlled manner. Extracellular vesicles have recently been reported both as immune activators and immune suppressors as they contain for example MHC class I and II and T cell co-stimulatory molecules (128, 129). However, the most described function of extracellular vesicles is triggering of the immune system, and very recently involvement of extracellular vesicles in inflammation resolution, tissue repair and regeneration was reported (130, 131).

## Impaired Resolution of Chronic Inflammation in the Intestine and the Lung

The pathways involved in the initiation of IBD or asthma differ from each other with respect to cytokine involvement and composition of the resident tissue. IBD is associated with a Th1/Th17 T cell-mediated response induced by interleukin-12 (IL-12) and IL-23, with concomitant increased production of IL-2, IL-17, IL-18, and IFN-γ (132, 133), whereas asthma and allergic diseases are associated with a typical T helper type 2 (Th2)-mediated response characterized by the production of interleukin-4 (IL-4), IL-5, and IL-13 (134). Therefore, specific tissue resolution processes exist, guided by the local microenvironment that are impaired in disease [reviewed recently in (135)]. There is increasing evidence that poor and/or inappropriate resolution of inflammation participates in the pathogenesis of IBD or asthma, being responsible in the long run for excessive tissue damage and pathology. In this chapter, we give some insights into resolution deficiencies in IBD and chronic asthma [for a recent and full review see (6, 136)].

### Inflammatory Bowel Diseases

IBD afflicts around 0.5% of the population in westernized countries (137). It is a chronic relapsing disease that includes Crohn's disease, a chronic trans-mural inflammatory disease of the gastrointestinal tract, mainly affecting the ileum and colon, characterized by leukocyte infiltration, granuloma, scarring, and fistulae and ulcerative colitis, a more superficial neutrophilic inflammatory lesion of the colon that progresses proximally. The inflammation partially, but never completely, resolves leading to tissue remodeling and disruption of the normal epithelial architecture that fails to fully regenerate, resulting in persistent increased epithelial permeability and inflammation.

In IBD multiple factors are identified that contribute to disease pathogenesis with a focus on host susceptibility genetic factors in combination with a qualitatively and quantitatively abnormal gut microbiota and an excessive immune response (138–142). The pro-inflammatory response is extensively studied, and the suppression of this phase is the main therapeutic strategy in Crohn's disease, and is still the central research focus, whereas much less is known about the resolution phase. Standard Crohn's disease therapy involves corticosteroids and immunosuppressants like azathioprine but these therapies are palliative and do not alter the natural history of IBD (143). In the last 20 years biological therapies (antibodies directed against cytokines, like anti-TNFα antibodies) have changed the treatment of more severe IBD. However, only ~50% of Crohn's Disease patients achieve clinical remission with the anti-TNFα *Humira*® or *Remicade*®. Indeed, the treatment results in a waning of the responsiveness to anti-TNF-α with time and only a minority of patients achieve mucosal healing (143, 144). Alternative therapies such as blocking the migration of effector T cells into the inflamed gut by targeting the α4β7 integrin, and recently also the blockade of IL-23 are showing additional success (145, 146) although they are effective only in a minority of patients. These treatments specifically blocking pro-inflammatory mediators cause immuno-suppression, and thereby induce an increased risk of infection. This exemplifies why new approaches and new therapies are needed to tackle the problems of chronic intestinal inflammation. Therefore, a better understanding of IBD pathophysiology is needed with a focus on the disturbed resolution of inflammation. In the line of this, the results of a recent meta-analysis focusing on mucosal healing in IBD as reported from endoscopic studies show that both partial, and full mucosal healing—thus a proper resolution of inflammation—predict favorable clinical outcome (147).

There is more and more evidence that persistent inflammation in IBD can occur as a result of inadequate engagement of a series of pro-resolving pathways and many studies have shown that pro-resolving mediators are able to prevent experimental colitis in different murine models [reviewed recently in (136, 148)].

MaR1 improves established chronic colitis induced by multiple dextran sulfate sodium (DSS) administrations (149). Systemic treatment of mice with PD1 or RvD5 protects against colitis and intestinal ischemia/reperfusion-induced inflammation (150). Other studies report protective roles for SPMs in experimental colitis [summarized in (151)]. Indications that SPM biosynthesis might be dysregulated in patients with IBD come from a study in which RvD5 and PD1 were upregulated in human IBD colon biopsies (150). Interestingly, mucosal expression of LXA_4_ is elevated exclusively in biopsies from individuals in remission from ulcerative colitis (152). Evidence, whether a defect in SPM signaling exists in IBD remains to be explored.

AnxA1 stimulates intestinal mucosal wound repair in a murine model of colitis (153) and AnxA1-containing exosomes and microparticles have been shown to accelerate the process of mucosal healing *in vivo* DSS models of colitis (154). In humans, AnxA1 is released by inflamed colonic biopsies from patients having ulcerative colitis (UC) and depends on the severity of inflammation (152, 155). In Crohn's disease, AnxA1 biosynthesis is dysregulated and higher levels correlate with successful intervention with biologicals against TNF-α (156). In another study in Crohn's Disease, AnxA1 is involved in intestinal homeostasis after anti-TNF-α treatment and suggested as a potential biomarker of therapeutic efficacy of anti-TNF-α treatment (157).

The production of IL-10 by Tregs is of particular interest in IBD. IL-10 deficiency in mice can lead to the development of spontaneous inflammatory bowel disease (158) and IL-10 receptor mutations found in patients result in an early-onset enterocolitis (159, 160). Furthermore, a IBD-like colitis can occur in response to recent immune checkpoint inhibitor treatments used in antitumor therapy aiming at blocking Treg cells (161). Tregs accumulate and IL-10 is upregulated in the gut during active IBD (162–166) but a clear demonstration that this pro-resolving mechanism operates in the gut mucosa in IBD is still missing.

Several authors report conflicting data whether or not it might be possible to use Galectin family member levels as markers for disease activity (167–171).

There is also evidence that α-MSH has potent anti-inflammatory activity in experimentally induced colitis (172, 173). Oral delivery via Bifidobacterium expressing α-melanocyte-stimulating hormone can prevent colitis in an experimental murine model (174).

H_2_S is able to improve the colonic barrier integrity in a murine model of experimental colitis (175). Administration of inhibitors of H_2_S synthesis in models of colitis result in an increase in severity of disease (176). In patients with active ulcerative colitis, alterations in the expression of genes involved in the purine metabolic pathway have been demonstrated (177). Like H_2_S, CO has been shown to exert potent protective effects in the gastro-intestinal tract (178).

Several gastro-intestinal neuroendocrine peptides and amines with pro-resolving properties, as members of the chromogranin/secretogranin family, VIP, somatostatin, and ghrelin are affected in experimental colitis and changes of these mediators occur during active IBD in patients [recently reviewed in (179)]. The exact role of neuroendocrine peptides/amines with pro-resolving properties in IBD has to be further elucidated.

### Asthma and Allergic Diseases

In the industrialized world, millions of individuals suffer from inappropriate activation and dysregulation of Th2 cell immune responses responsible for allergic asthma and rhinitis, food allergies and atopic dermatitis (also known as eczema), being part of a process called the atopic march. These disorders are increasingly prevalent and are a major public health problem (180). Th2 cell mediated immune responses are characterized by the release of type 2 signature cytokines (i.e., IL-4, IL-5, IL-9, and IL-13) from cells of both the innate and adaptive immune systems (134, 181).

Current therapeutic strategies for chronic Th2 immune disorders are mainly anti-inflammatory, and aim at controlling symptoms. In chronic persistent asthma, inhaled corticosteroids are the main anti-inflammatory treatment effective in most patients, causing relatively minor adverse effects (182). A subset of asthma patients (~10%) experience persistent symptoms and/or frequent exacerbations despite high doses of inhaled corticosteroids and are often treated with prolonged systemic corticotherapy having many potential side-effects (183). Monoclonal antibodies targeting inflammatory pathways that activate immune responses leading to airway inflammation have been developed to help broaden the current arsenal of asthma treatment options (184). The first anti-body based biological therapy approved for treatment of asthma was omalizumab, targeting IgE, a component of the allergic cascade (185). More recently, monoclonal antibodies have been approved, targeting IL-5 or its receptor (mepolizumab, reslizumab, benralizumab), a key cytokine promoting eosinophil inflammation (186). Other monoclonal antibodies targeting a wide variety of intermediaries in the pro-inflammatory cascade are currently being tested for their effectiveness in the treatment of asthma (184). These biological therapies can reduce exacerbations and have glucocorticoid-sparing effects, but the clinical responses to these antibody-therapies are variable, with at least 30% of severe asthmatic patients being non-responders (187).

These therapeutic strategies can be combined with allergen-specific immunotherapies in chronic allergic diseases that are able to improve symptoms but they do not cure allergic disorders (188, 189).

As for IBD, there is increasing evidence that chronic and uncontrolled inflammation in Th2 immune disorders, might result not just from an excessive uncontrolled pro-inflammatory response but also from uncontrolled and insufficient engagement of pro-resolving pathways and thus impaired resolution of exacerbations (5, 33, 190).

First, pro-resolving mediators have proved efficient at improving disease and inflammatory outcomes in a variety of asthma models. Treatment with SPMs decreases key features of asthma pathobiology, including airway hyperresponsiveness, mucus metaplasia, and Th2 cell bronchial inflammation (53, 191–193). AnxA1 deficient mice exhibit spontaneous airway hyperresponsiveness and exacerbated allergen responses (194) and AnxA1 mimetics inhibit eosinophil recruitment (195). Ablation of IL-10 signaling in Th2 cells leads to exacerbated pulmonary inflammation (196). In murine models, IL10 knock-out mice develop enhanced allergic airway responses (197, 198) [(199) #1421]. IL-10 also inhibits pro-inflammatory cytokine production by Th2 cells and down-regulates mast cell and eosinophil function (200). Administration of recombinant Gal-9 or α-MSH diminish allergic airway inflammation [(201) #1453; (202)]. Low H_2_S production in ovalbumin sensitized and challenged mice results in aggravated AHR and increased airway inflammation (203). In a rat model of asthma, exogenous administration of H_2_S reduces airway inflammation and airway remodeling (204). VIP can inhibit eosinophil migration (205) and airway remodeling in asthmatic mice (206). Intestinal epithelial cell-derived Gal-9 is involved in the resolution of allergic responses through the induction of tolerogenic DCs and associated Treg cell response (207, 208). In addition, some of these galectins can block IgE binding on mast cells and as such, inhibit allergic inflammation (209, 210). The role of adenosine in the resolution of inflammation of chronic asthma is not yet elucidated.

Based on the evidence for the functional importance of pro-resolving mediators in allergic asthma mouse models, defects in the production or the activity of pro-resolving mediators might therefore participate in the chronicity and severity of human asthma. Several studies in distinct populations have reported that SPMs are underproduced in more severe asthma together with a defect in the expression of their related receptors [for a recent review see (6)]. Annexin A1 (AnxA1) levels are also decreased in patients with asthma (211), and in wheezy infants (212). Moreover, plasma and bronchoalveolar AnxA1 levels are correlated with lung function (FEV1 %) (213, 214). Compared with non-asthmatics, asthmatic individuals have reduced levels of IL- 10 in bronchoalveolar lavage fluid (215) and a decreased secretion of IL-10 from alveolar macrophages (216).

Furthermore, polymorphisms in the IL10 gene resulting in low IL-10 production have been associated with severe asthma (217). T cells from allergic asthmatic patients are partially resistant to IL-10 mediated suppression [(218) #1396]. In humans, galectin-3 production has been reported to be lower in asthma, particularly in neutrophilic asthma (76, 219). Macrophages from sputum samples of asthma patients express reduced levels of Gal-1 and Gal-9 (82). Several human studies have shown a decrease in serum or exhaled-breath H_2_S levels in both adult and infants (220–222). Moreover, lower H_2_S levels are correlated with abnormal pulmonary lung function tests and severity of asthma (220, 221). There is relatively limited information available regarding the role of neuropeptides in the resolution of inflammation in asthma but Tomaki et al. found that SubP levels in sputum correlate with airway obstruction in asthma (223).

## How to Improve Resolution in Chronic Inflammatory Diseases

Targeting the inflammation phase has been the main focus in medical research for the past decades, resulting in treatment options for immune-mediated diseases that dampen inflammation and display immunosuppressive actions (see Figure 1). This comes with a burden for the body, since anti-inflammatory immune suppressive therapies, for example corticosteroids or anti-TNF inhibitors, may have increased risks of infection. In addition, the development of expensive targeted anti-inflammatory biologicals creates an economic burden on society.

Immune responses are very complex and only recently the first initiative to define the naturally occurring variation and the boundaries of a healthy immune response to complex stimuli was published (224). Interestingly, there is considerable variation in the ability of tissue inflammation to resolve within a healthy population (225).

Different endogenous factors such as age, genetics, sex, ethnicity, might influence the nature and extent of the acute inflammatory response including the resolution process (226). Studies have highlighted how epigenetic reprogramming can lead to chronic inflammation and impede resolution resulting in inflammatory diseases (227, 228). Gut microflora plays a critical role in the stimulation and maturation of a balanced immune system (229). There is also evidence that life style factors, physical and psychological conditions can impact on the magnitude of the inflammatory response (230).

**Figure 5:**
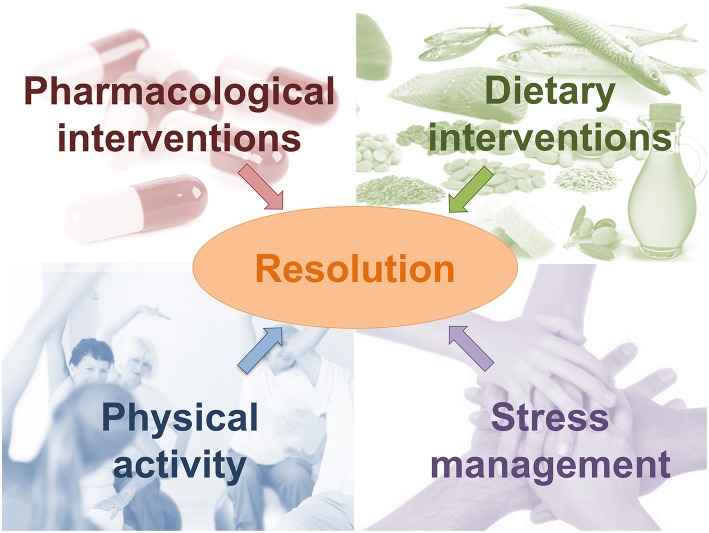
New resolution-based strategies able to direct the inflammatory processes in a controlled way.

Understanding the mechanism required for adequate resolution of inflammation may support the development of new resolution-based strategies able to direct the inflammatory processes in a controlled way. Different approaches can be considered (Figure 5).

### Pharmacological Interventions

Many current therapeutic approaches to manage chronic inflammation aim at repressing overactive pro-inflammatory responses by reducing pro-inflammatory mediator activity (i.e., corticosteroids or biologics). In addition to their potent anti-inflammatory properties, steroids can display several pro-resolving properties (7, 65, 231, 232), however, this comes with many potential side-effects, such as osteoporosis, diabetes, systemic hypertension, and impaired immune function.

Several synthetic pharmaceutical analogs with pro-resolving properties have been proven to be active in animal models [reviewed in (233, 234)]. Toward this end, stable synthetic mimetics to endogenous SPMs are under development, and in matching studies, these mimetics display similar biological actions to the parent mediators in animal models of diverse inflammatory disorders with an advantage of resisting local inactivation (including mimetics encapsulated in vesicles) (235–239). Several of these mimetics are in pre-clinical development programs for different chronic inflammatory conditions [for reviews see (3, 240)]. In a double-blind placebo-controlled clinical trial, a topical 15-R/S-methyl-LXA_4_ preparation was tested for the treatment of infantile atopic eczema (241). In this study, the efficacy of the lipoxin mimetic was at least equivalent to gold standard topical steroid therapy for the reduction of eczema severity by quantitative and qualitative measures.

### Dietary Interventions

#### Omega-3 PUFAs

The main bioactive omega−3 PUFAs, eicosapentaenoic acid (EPA) and docosahexaenoic acid (DHA), are poorly synthesized in humans. They are components of seafood, especially oily fish, of fish oil, liver oil, krill oil and algal oil supplements, and of a small number of highly concentrated pseudo-pharmaceutical products.

EPA and DHA have long been known to have beneficial health effects including anti-inflammatory, anti-thrombotic, and immuno-regulatory properties (242–244). These n−3 LCPUFAs are substrates for biosynthesis of potent SPMs such as resolvins, protectins, and maresins (Figure 3). DHA is concentrated in neural tissues including brain and retina and in sperm; EPA and DHA are found in membranes of all other cells and tissues and in human milk (245–247). Increased dietary intake of EPA and DHA results in their enrichment in blood and in many cells and tissues. Omega-3 PUFAs can exert significant effects on the intestinal environment and modulate the gut microbiota composition (248).

The airway mucosa is also enriched with DHA in healthy individuals (249). Interestingly, airway mucosal levels of n−3 PUFAs are lower in patients with asthma than in people without asthma (249). Population surveys report that diets rich in n-3 fatty acids are associated with lower asthma prevalence (250).

It is noteworthy that SPMs are present at significant levels in placenta and human milk (251–253), which suggests an important role for SPMs in health maintenance during a particularly vulnerable period of infant development. In a recent randomized placebo-controlled study from a Danish birth cohort, supplementation with a high dose of n-3-LCPUFAs (a dose corresponding to a 10–20-times increase of the normal intake) during the third trimester of pregnancy was associated with a significantly lower risk of asthma symptoms and fewer respiratory infections in children at 3 years (254). This effect was most prominent among children of women who had low pre-intervention EPA and DHA blood levels (255).

Recent human studies have shown that increased intake of EPA and DHA results in higher concentrations of selected SPMs in the bloodstream (256–258). High doses of n-3 PUFAs reduce pain and other symptoms in patients with rheumatoid arthritis (259, 260). Many of the mechanisms of action of EPA and DHA suggest that they reduce the pro-inflammatory response (242, 243). However, the discovery of SPMs derived from EPA and DHA and the potency of those SPMs in animal models (see earlier) hints that their main action might be promotion of resolution.

#### Pre- and Probiotics

The prebiotic galacto- and fructo-oligosaccharides, so-called non-digestible oligosaccharides, in combination with probiotic bacteria induced the resolution-inducing lectin galectin 9 in mouse models for food allergy and in infants suffering from cow's milk allergy (209). *In vitro* studies showed that the epithelial release of gal 9 by this specific combination of pre- and probiotic induces tolerogenic DCs that in turn upregulated Treg cells (209, 261). In addition, the combination of *Bifidobacterium longum* with inulin-oligofructose resulted in resolution of inflammation in patients suffering from active colitis (262, 263). Several preclinical studies have demonstrated that treatment with specific bacterial strains induces an IL-10 response associated with a faster resolution of inflammation in allergy and Crohn's Disease models (264–266). Overall, there are some indications that dietary intervention with pre- and probiotics promotes the induction of resolution of inflammation. However, the exact mechanisms of resolution induced by pre- and probiotics remains to be examined.

### Exercise/Physical Activity

Exercise enhances functional capacity, through increased aerobic capacity and muscle strength, improves quality of life and has the potential to protect from cardiovascular disease, type 2 diabetes mellitus, and certain types of cancer [reviewed in (267)]. The potential mechanisms underlying exercise-mediated protection toward these disorders, include changes in body composition, neuro-hormonal status, as well as effects on resolution pathways.

Indeed, acute increases in intramuscular IL-6 following exercise promote resolution processes by increasing the synthesis of anti-inflammatory cytokines such as IL-1RA and IL-10, and inhibit pro-inflammatory cytokines such as TNF-α (268). Exercise also modulates the production of the PUFA derived SPMs described earlier. Indeed, maximal physical exertion was found to result in a rapid post-exercise increase in the urinary excretion of arachidonic acid (AA) derived lipoxin A_4_ (LXA_4_) in healthy subjects (269). Similarly, EPA derived resolvin E1 (RvE1) transiently increases early in human serum following exercise and DHA derived resolvin D1 (RvD1) and protectins increased later during recovery (270).

Interestingly, levels of LXA_4_ are found to increase immediately after exercise in exhaled air condensate of asthmatic children with exercise induced bronchoconstriction (EIB) (271). The authors hypothesized that airway LXA_4_ increases to compensate bronchoconstriction and to suppress acute inflammation, and that spontaneous bronchodilation after EIB may be due to LXA_4_. In murine studies in relation to asthma, physical exercise reduced asthma associated bronchial inflammation (IL-4, IL-5 expression and eosinophil infiltrate) which was associated with an increase of IL-10 (272, 273). Exercise in animal models of colitis also reduced levels of TNF-α, and decreased markers of oxidative stress and histological damage to the colon in parallel to increased levels of the resolution-promoting and anti-inflammatory cytokine IL-10 (274).

### Stress Management

Emotional, cognitive, and psychosocial factors are now widely recognized as significant determinants of health outcomes including impacts on the immune system (275). In this sense, a broad variety of mind-body therapies that are able to decrease stress, including meditation-based stress reduction programs (MBSR) and yoga have been increasingly proposed over the past years, as substantial adjuncts to conventional medical treatment in chronic inflammatory diseases and cancer patients (275). Convergent evidence suggests that these mind-body therapies may have effects on immune functions including effects on the hypothalamic-pituitary-adrenocortical (HPA) axis function (276) and on NK cell functions and IL-10 levels within patients suffering from chronic inflammatory disorders (277–279). More precise impacts of the mental state on resolution parameters remain to be examined.

## Conclusion

There are a number of immune-mediated chronic diseases that might, at least in part, be controlled or prevented in an immune fit person, including IBD, allergy, and asthma developed in this review, as well as rheumatoid arthritis, chronic obstructive pulmonary disease (COPD), Parkinson's disease, Alzheimer's disease, multiple sclerosis, diabetes or myalgic encephalomyelitis. The focus of research on resolution of the immune response as a possible therapeutic approach has only been apparent in the twenty-first century and there is now increasing evidence that poor and/or inappropriate resolution of inflammation participates in the pathogenesis of IBD or asthma, being responsible in the long run for excessive tissue damage and pathology. This might now open a whole area in the development of personalized therapeutic options for chronic immune diseases driven in part by maladaptive, non-resolving inflammation.

Moreover, since there is a strong link between a compromised immune system and the brain, individuals can experience a reduced quality of life and lack of well-being (280, 281). Many chronic inflammatory diseases are associated with depression, anxiety, and reduced cognitive function (282) and it is becoming apparent that many brain diseases (psychiatric and neurological) are associated with activation of the immune system (281, 283). Therefore, deviant immune fitness because of overreaction and poor or defective resolution of the immune system has an enormous impact and is a central issue in these chronic immune diseases.

## Author Contributions

All authors listed have made a substantial, direct and intellectual contribution to the work, and approved it for publication.

### Conflict of Interest Statement

The authors declare that the research was conducted in the absence of any commercial or financial relationships that could be construed as a potential conflict of interest.
